# Evaluation of Etiology, Clinical Manifestations, Diagnosis, Follow-up, Histopathology and Prognosis Factors in Papillary Thyroid Microcarcinoma: A Systematic Review and Meta-analysis

**DOI:** 10.30699/IJP.2023.2005196.3134

**Published:** 2023-10-15

**Authors:** Shiva Didehban, Alireza Abdollahi, Alipasha Meysamie

**Affiliations:** 1 *Department of Pathology, School of Medicine, Tehran University of Medical Sciences, Tehran, Iran*; 2 *Thrombosis Hemostasis Research Center, Tehran University of Medical Sciences, Tehran, Iran*; 3 *Department of Community Medicine, School of Medicine, Tehran University of Medical Sciences, Tehran, Iran*

**Keywords:** Clinical manifestation, Etiology, Histopathology, Meta-analysis, Papillary thyroid microcarcinoma

## Abstract

**Background & Objective:**

The most frequent type of cancer found in the endocrine system is thyroid carcinoma. Among well-differentiated thyroid malignancies, the most commonly occurring type is identified as papillary thyroid carcinoma (PTC), which makes up 70-90% of the cases. A subtype of PTC is papillary thyroid microcarcinoma (PTMC), which includes tumors smaller than 10 mm in diameter. Due to the advancements in diagnostic techniques, the incidence of this type of cancer is on the rise. In this study, we aimed to analyze the factors worsening the PTMC prognosis.

**Methods:**

In the first step, we searched various databases for the factors affecting this tumor. The relevant articles were collected and different outcomes of this tumor and its associated factors which were studied in more than one article, were classified. Finally, we conducted a meta-analysis of these outcomes and their related factors.

**Results:**

In the meta-analysis, a significantly association was found between the following factors: recurrence with gender (*P*<0.001) lymph node metastasis (LNM) (*P*= 0.003), and extrathyroidal invasion (*P*<0.001); lymph node metastasis with extrathyroidal invasion (*P*<0.001), and multifocality (*P*<0.001); central lymph node metastasis (CLNM) with gender (*P*=0.001), tumor size (*P*<0.001), extracapsular invasion (*P*<0.001), lateral cervical lymph node metastasis (*P*<0.001), and extrathyroidal invasion (*P*<0.001); lymph node metastasis resulted in poor outcomes (*P*<0.001); and finally tumor size with BRAFV600E mutation (*P*<0.001).

**Conclusion:**

**In conclusion, it is essential to note that greater awareness and understanding of this tumor characteristics and special and separate attention to PTMC can significantly improve the society overall health**.

## Introduction

The most frequent cancer found in the endocrine system is thyroid carcinoma, with an annual occurrence rate of approximately 9 cases per 100,000 individuals. These cancers can vary widely in their behavior, ranging from small and unimportant microcarcinomas found by chance to practically incurable and aggressive anaplastic carcinomas (1, 2).

The most prevalent form of all well-differentiated thyroid cancers is papillary thyroid carcinoma (PTC), representing 70-90% of cases. Papillary thyroid carcinoma can affect children, even though the typical age of diagnosis for this malignancy is 45 years, and the likelihood of its occurrence rises with age (1, 2). The World Health Organization (WHO) has established a distinct subgroup within the PTC category, known as papillary thyroid microcarcinoma (PTMC), based on the largest dimension that does not exceed 1.0 cm. PTMC is often not identifiable during clinical examination, instead it is found as a byproduct of examining thyroid tissue samples after surgery for non-cancerous thyroid conditions or post-mortem examinations. To make a diagnosis, characteristic cytological features of PTC, such as the presence of psammoma bodies and cleaved nuclei that give rise to the presence of enlarged nucleoli resulting in "Orphan Annie" appearance, and the formation of papillary structures, can be identified through the fine-needle aspiration (FNA) or surgical resection (2, 3). Furthermore, the advancements in the precision of pathological evaluations, especially with the number and thickness of the anatomical slices obtained from the thyroid tissue specimens, have resulted in an increased incidence of incidental PTMC diagnosis (3-7).

The rates of thyroid cancer have been on a steady rise globally (8, 9), including an increase in the number of PTMC cases due to better diagnostic techniques (10, 11). This study aims to review previous studies on PTMC and their association with the factors that worsen tumor prognosis through a meta-analysis. The objective is to raise awareness among physicians about the characteristics of this tumor, enabling earlier diagnosis and treatment that could potentially enhance the PTMC patients’ survival and decrease the cancer-related death.

## Material and Methods

**Search Plan**

To initiate the systematic review, we searched for the articles about Papillary Thyroid Microcarcinoma (PTMC) using specific keywords such as etiology, histopathology, diagnosis, clinical presentation, follow-up, and prognosis. Searches in databases such as PubMed, Ovid, and Google Scholar were conducted with no language restrictions from January 1998 to January 2014. 

Exclusion criteria

Various criteria were employed to eliminate the articles from consideration in this study. We excluded articles that were: a) review articles on papillary thyroid microcarcinoma, b) PTMC review articles references, c) search results that were books, not original articles, d) identical articles that were published in various databases, e) articles that discussed thyroid tumors apart from PTMC, and f) papers that focused on features of PTMC not relevant to the present study. 

**Fig. 1:**
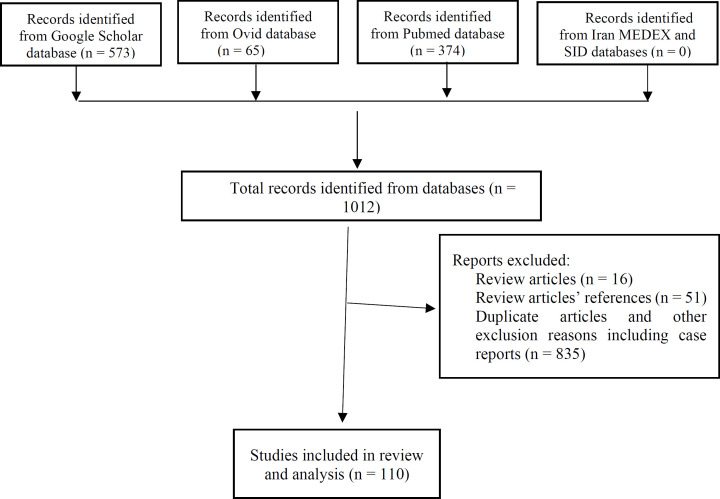
PRISMA flowchart of classification of articles from searches of databases for systematic review

Although we initially found 1012 articles, these criteria reduced the number of articles to 110 (Figure 1), then categorized these articles into six groups based on their titles and main subjects: Etiology (5 articles), prognosis (27 articles), diagnosis (19 articles), follow-up (20 articles), histopathology (14 articles), and clinical presentation (25 articles). The references of all the articles were checked. Due to the wide scope utilized in the primary search, all articles mentioned in the references that did not meet the exclusion criteria were included in the primary search results.

**Data Extraction**

In this section, for analyzing the relationships between some outcomes and various factors, e.g., lymph node metastasis and patient's age, a table was formed in Excel 2016, and each article data were entered in one sheet. In this table, 16 sheets with following titles were created: Extrathyroidal extension, Central lymph node metastasis (CLNM), Bad outcome, Occult carcinoma of the contralateral lobe, false positivity, BRAF^V600E^ mutation, Contralateral central lymph node metastasis (CCLNM), Recurrence, Radiation effect on PTMC, Absence of BRAF^V600E^ mutation, Lymph node metastasis (LNM), Bilateral PTMC, Malignancy rate, Size enlargement, Turning into clinical disease, and Lateral lymph node metastasis.

In each sheet, the articles were sited in rows and columns consisted of the analyzed variables: gender, age>45 years, ≤45 years, >50 years, ≤50 years, mean age of years, the size of tumor >5mm, ≤5 mm, >7 mm, ≤7 mm, >8 mm, ≤8 mm, preoperative size of tumor>7 mm, ≤7 mm, the mean size of tumor in mm, ultrasound (US) tumor size in mm, FDG visual uptake positivity, FDG SUVs, MACIS (metastasis, age, completeness, invasiveness, size) score ˂6, ≥6, lymphovascular invasion, pathological involvement of ipsilateral central lymph node, TNM tumor stage, extracapsular extension, bilateral involvement, lateral cervical lymph node metastasis, multifocality in unilateral lobe, presence of preoperative contralateral benign nodule, stages 1, 2, 3, and 4, extrathyroidal extension, extrathyroidal extension on US, multifocality, preoperative multifocality, distant metastasis, type of surgery (total thyroidectomy vs. lobectomy), lymph node metastasis, BRAF^V600E^ mutation, radiation dose ˂5 mGy, 5-100 mGy, 100-500 mGy, ˃500 mGy, follicular variant of tumor, central lymph node metastasis, mean platelet number, tumor location in upper third, in middle, or lower third, calcification, bilaterality on US, preoperative bilaterality, I^131^ therapy, hard malignancy on Rago score, CLNM on US, lateral lymph node metastasis on US, S100A4 expression, and not well-defined margin on US.

For each variable, these indexes were considered: relative frequency for the qualitative variables, mean for the quantitative variables, the total number of patients in each group, the upper limit of 95% confidence interval (CI), and standard error. 

In the last step, we classified the factors associated with each outcome discussed in more than one article. Every outcome was as an Excel 2016 file in which the factors were sorted sheet by sheet. Rows of each sheet contained articles, and the columns were these indexes: frequency of patients positive for the outcome and studied factor, negative for the outcome but positive for the studied factor, positive for the outcome but negative for the studied factor, negative for the outcome and studied factor, total number of patients with the mentioned outcome, the upper limit of 95% CI and the standard error for the factor. Finally, six tables were formed with these titles: Bad outcome, BRAF^V600E^ mutation, Central lymph node metastasis, Extrathyroidal extension, Lymph node metastasis, and Recurrence.

**Data Analysis**

To meta-analyzing the articles outcomes attributable to disease according to different variables (such as gender, age, tumor size, lymph node involvement, tumor stage, extrathyroidal extension, multifocality, extracapsular invasion, lateral cervical lymph node involvement, and bilaterality), the STATA 15.0 software was used. Chi-squared (chi-2) test was done to assess the homogeneity across the articles. If the results of the articles showed homogeneity, we used a fixed-effect model and assessed using the I2 test. If the results were heterogeneous, we conducted a Tau-squared analysis and considered a random-effect model. "Pooled RR" was computed according to the weighting articles on the basis of their sample size. The P-value less than 0.05 was considered statistically significant. A forest plot was drawn for the articles Relative Risk (RR) and Pooled RR.

## Results

The articles assessing the same outcome were analyzed for their factors. The details of each article and meta-analysis results are explained below and in Table 1. If there was no heterogeneity, the fixed-effect logistic regression method was utilized, and random-effect model analysis was used if heterogeneity was seen across the articles.

**Table 1:** Relationship between the outcome and some factors

No.	Outcome	Factor	No. of articles	Outcome (+)		Outcome (-)		Pooled RR	95% CI	P value
1	**Recurrence**	Gender	2	Male=26 Male=7	Female=28 Female=21	Male=197 Male=23	Female=726 Female=98	2.416	1.594-3.663	<0.001
2		Age	2	>45 yrs=34 >45 yrs=15	≤45 yrs=20 ≤45 yrs=13	>45 yrs=498 >45 yrs=68	≤45 yrs=425 ≤45 yrs=53	1.221	0.803-1.855	0.351
3		Size	2	>5 mm=21 >5 mm=10	≤5 mm=33 ≤5 mm=18	>5 mm=324 >5 mm=47	≤5 mm=599 ≤5 mm=74	1.066	0.698-1.626	0.768
4		LNM	4	Present=19 Present=10 Present=19 Present=13	Absent=35 Absent=18 Absent=21 Absent=4	Present=210 Present=5 Present=134 Present=99	Absent=713 Absent=116 Absent=231 Absent=273	2.967	1.432-6.147	0.003
5		TNM	2	High=24 High=8	Low=30 Low=2	High=261 High=10	Low=54 Low=10	3.714	0.797-17.301	0.095
6		EE	3	Present=40 Present=17 Present=10	Absent=14 Absent=11 Absent=7	Present=245 Present=2 Present=120	Absent=678 Absent=119 Absent=252	6.366	4.278-9.475	<0.001
7		Multifocality	2	Multifocal=25 Multifocal=18	Unifocal=29 Unifocal=10	Multifocal=298 Multifocal=17	Unifocal=625 Unifocal=104	3.137	0.950-10.361	0.061
8	**LNM**	EE	2	Present=40 Present=8	Absent=34 Absent=9	Present=30 Present=6	Absent=94 Absent=38	2.273	1.654-3.124	<0.001
9		Multifocality	2	Multifocal=34 Multifocal=37	Unifocal=40 Unifocal=42	Multifocal=30 Multifocal=35	Unifocal=94 Unifocal=98	1.745	1.369-2.223	<0.001
10	**Extrathyroidal Extension**	Gender	2	Male=9 Male=7	Female=35 Female=58	Male=8 Male=5	Female=35 Female=56	1.098	0.766-1.575	0.611
11	**Bad outcome**	LNM	2	Present=14 Present=3	Absent=7 Absent=6	Present=31 Present=5	Absent=127 Absent=83	5.869	2.890-11.922	<0.001
12	**Central lymph node metastasis**	Gender	6	Male=8 Male=18 Male=44 Male=9 Male=13 Male=18	Female=19 Female=70 Female=16 Female=25 Female=48 Female=121	Male=9 Male=23 Male=16 Male=3 Male=6 Male=50	Female=51 Female=224 Female=46 Female=89 Female=93 Female=294	1.955	1.333-2.869	0.001
13		Age	2	>45 yrs=20 >45 yrs=45	≤45 yrs=7 ≤45 yrs=43	>45 yrs=40 >45 yrs=161	≤45 yrs=20 ≤45 yrs=86	0.753	0.548-1.033	0.079
14		Size	7	>5 mm=16 >5 mm=72 >5 mm=55 >5 mm=99 >5 mm=15 >5 mm=159 >5 mm=56	≤5 mm=11 ≤5 mm=16 ≤5 mm=6 ≤5 mm=40 ≤5 mm=1 ≤5 mm=59 ≤5 mm=19	>5 mm=28 >5 mm=138 >5 mm=64 >5 mm=171 >5 mm=11 >5 mm=357 >5 mm=53	≤5 mm=32 ≤5 mm=109 ≤5 mm=35 ≤5 mm=173 ≤5 mm=25 ≤5 mm=267 ≤5 mm=33	1.954	1.664-2.296	<0.001
15		ECS	5	Present=62 Present=90 Present=11 Present=129 Present=39	Absent=26 Absent=49 Absent=5 Absent=89 Absent=36	Present=125 Present=154 Present=2 Present=278 Present=37	Absent=122 Absent=190 Absent=34 Absent=346 Absent=49	1.802	1.325-2.452	<0.001
16		Bilaterality	3	Present=26 Present=32 Present=26	Absent=34 Absent=107 Absent=49	Present=34 Present=37 Present=11	Absent=28 Absent=307 Absent=75	1.375	0.836-2.263	0.210
17		LCLNM	3	Present=17 Present=8 Present=13	Absent=122 Absent=8 Absent=62	Present=3 Present=1 Present=2	Absent=341 Absent=35 Absent=84	2.941	1.931-4.478	<0.001
18		EE	2	Present=48 Present=38	Absent=40 Absent=23	Present=73 Present=44	Absent=174 Absent=55	1.875	1.432-2.454	<0.001
19	**BRAF** ^V600E^ ** Mutation**	Gender	2	Male=118 Male=30	Female=274 Female=183	Male=105 Male=14	Female=480 Female=112	1.279	0.968-1.691	0.084
20		Size	2	>5 mm=165 >5 mm=132	≤ 5mm=227 ≤ 5mm=81	>5 mm=180 >5 mm=46	≤ 5mm=405 ≤ 5mm=80	1.381	1.230-1.551	<0.001
21		LNM	3	Present=199 Present=72 Present=19	Absent=193 Absent=141 Absent=15	Present=30 Present=37 Present=	Absent=555 Absent=89 Absent=15	1.905	0.833-4.357	0.127
22		TNM	2	High=173 High=67	Low=219 Low=146	High=112 High=39	Low=473 Low=87	1.394	0.739-2.628	0.305
23		Multifocality	2	Multifocal=127 Multifocal=67	Unifocal=265 Unifocal=146	Multifocal=196 Multifocal=28	Unifocal=389 Unifocal=98	1.037	0.918-1.171	0.563

**Gender and Recurrence**

Two articles (12, 13) were analyzed in this part. Because of the lack of heterogeneity (heterogeneity χ^2^= 3.45, *P*=0.063), the fixed-effect logistic regression model was used to report the significant results. 

RR1= 3.140; 95% CI= 1.881-5.241, RR2= 1.322; 95% CI= 0.621-2.816

Pooled RR= 2.416; 95% CI = 1.594-3.663,* P*=0.000 

**Age and Recurrence**

In the two articles (12, 13), there was no heterogeneity across the studies (heterogeneity χ2= 1.01, *P*=0.315):

RR1= 1.422; 95% CI= 0.831-2.435, RR2= 0.918; 95% CI= 0.470-1.791

Pooled RR=1.221; 95% CI= 0.803-1.855, *P*=0.351

**Tumor Size and Recurrence**

Two articles (12, 13) used to analyze this association were not heterogeneous (heterogeneity χ^2^= 0.34, *P*=0.557). The results of the analysis were not significant.

RR1= 1.166; 95% CI= 0.685-1.983, RR2= 0.897; 95% CI= 0.446-1.804

Pooled RR= 1.066; 95% CI= 0.698-1.626, *P*= 0.768

**Lymph Node Metastasis and Recurrence**

Four articles (12-15) were used to conduct this analysis, and this relationship was not heterogeneous (heterogeneity χ^2^= 15.09; *P*=0.002):

RR1= 1.773; 95% CI= 1.035-3.038, RR2= 4.963; 95% CI= 2.837- 8.682

RR3= 1.490; 95% CI= 0.828-2.681, RR4= 8.038; 95% CI= 2.678-24.122

Pooled RR= 2.967; 95% CI= 1.432-6.147, *P*=0.003

**Tumor Stage and Recurrence**

In this section, two articles (12, 16) were analyzed by the random-effect model because of the articles heterogeneity (heterogeneity χ^2^= 4.14, *P*=0.042):

RR1=1.942; 95% CI= 1.156-3.263, RR2= 9.556; 95% CI= 2.245-40.675

Pooled RR= 3.714; 95% CI= 0.797-17.301, *P*=0.095

**Extrathyroidal Extension and Recurrence**

Three articles (12, 13, 15) were used to investigate this relationship. Analysis was done by the fixed-effect model because of homogeneity (heterogeneity χ^2^= 5.76, *P*=0.056):

RR1= 6.937; 95% CI= 3.835-12.550, RR2= 10.574; 95% CI= 5.885-19.00,

RR3= 2.846; 95% CI= 1.109-7.305

Pooled RR= 6.366; 95% CI= 4.278-9.475, *P*=0.000

**Multifocality and Recurrence**

There were two articles (12, 13) in this part. Since they were heterogeneous (heterogeneity χ2= 7.91, *P*=0.005), an analysis was conducted using the random-effect model: 

RR1= 1.745; 95% CI= 1.040-2.930, RR2= 5.863; 95% CI= 2.988-11.502

Pooled RR= 3.137; 95% CI= 0.950-10.361, *P*=0.061

**Extrathyroidal Extension and Lymph Node Metastasis**

Two articles (17, 18) had the criteria for the analysis in this part. Since there was no heterogeneity (heterogeneity χ2 = 0.61, *P* 0.435), the significant analysis output was obtained from the fixed-effect model:

RR1= 2.151; 95% CI= 1.512-3.060, RR2= 2.984; 95% CI= 1.421-6.268

Pooled RR= 2.273; 95% CI= 1.654-3.124, *P*=0.000

**Multifocality and Lmph Node Metastasis**

In this section, analysis was performed on two articles (17, 19). Since no heterogeneity was observed between them (heterogeneity χ^2^=0.02, *P*=0.877), a fixed-effect model was used for the analysis:

RR1= 1.780; 95% CI= 1.258-2.518, RR2= 1.713; 95% CI= 1.221-2.403

Pooled RR= 1.745; 95% CI= 1.369-2.223, *P*=0.000

**Gender and Extrathyroidal Extension**

Two articles (20, 21) were analyzed by the fixed-effect model because of their homogeneity (heterogeneity χ2= 0.05, *P*=0.828). The non-significant results are as below:

RR1= 1.059; 95% CI= 0.639-1.756, RR2= 1.147; 95% CI= 0.688-1.911

Pooled RR= 1.098; 95% CI= 0.766-1.575, *P*=0.611

**Gender and Central Lymph Node Metastasis**

Six articles (20-25) were analyzed in this part. As heterogeneity was seen across the articles (heterogeneity χ^2^= 21.76, *P*=0.001), an analysis was conducted using the random-effect model:

RR1= 1.734; 95% CI= 0.920-3.267, RR2= 1.844; 95% CI= 1.234-2.756

RR3= 2.842; 95% CI= 1.814-4.451, RR4= 3.420; 95% CI= 2.125- 5.505

RR5= 2.010; 95% CI= 1.371- 2.946, RR6= 0.908; 95% CI= 0.594-1.387

Pooled RR= 1.955; 95% CI= 1.333-2.869, *P*=0.001

**Age and Central Lymph Node Metastasis**

Two articles (20, 22) were analyzed for this relationship. Articles were not heterogeneous (heterogeneity χ2= 2.64, *P*=0.104), so the non-significant results of the analysis were obtained by the fixed-effect model:

RR1= 1.286; 95% CI= 0.619-2.671, RR2= 0.655; 95% CI= 0.459-0.935

Pooled RR= 0.753; 95% CI= 0.548-1.033, *P*=0.079

**Tumor size and Central Lymph Node Metastasis**

**Fig. 2:**
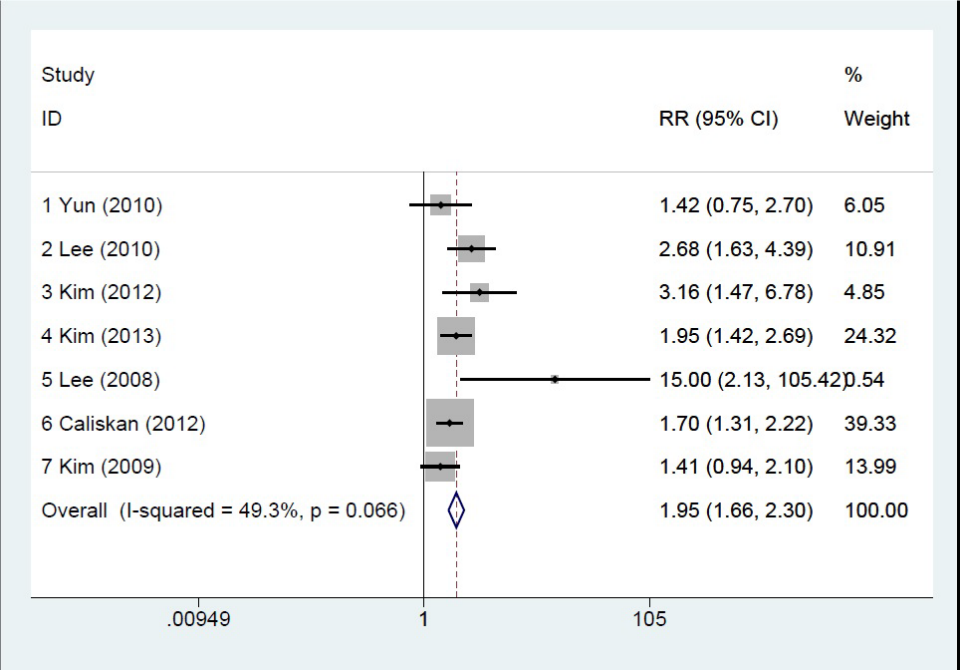
Forest plot of the tumor size and CLNM relationship

Seven articles (20, 22, 24-28) were assessed in this section (Figure 2). As the articles were not heterogeneous (heterogeneity χ^2^= 11.84, *P*=0.066), analysis was done by the fixed-effect model. The significant results are as follows:

RR1= 1.421; 95% CI= 0.748-2.702, RR2= 2.679; 95% CI= 1.634-4.392

RR3= 3.158; 95% CI= 1.471-6.782, RR4= 1.952; 95% CI= 1.417-2.690

RR5= 15.000; 95% CI= 2.134-105.418, RR6= 1.703; 95% CI= 1.307-2.218

RR7= 1.406; 95% CI= 0.941-2.102

Pooled RR= 1.954; 95% CI= 1.664-2.296, *P*=0.000

**Extracapsular Spread and Central Lymph Node Metastasis**

Five articles (22, 25-28) were assessed in this part. Analysis was done by the random-effect model because of heterogeneous articles (heterogeneity χ^2^= 14.61, *P*=0.006). Significant results of the analysis are as follows:

RR1= 1.887; 95% CI= 1.260-2.827, RR2= 1.799; 95% CI= 1.334-2.426

RR3= 6.600; 95% CI= 2.819-15.451, RR4= 1.549; 95% CI= 1.226-1.957

RR5= 1.212; 95% CI= 0.870-1.687

Pooled RR= 1.802; 95% CI= 1.325-2.452, *P*=0.000

**Bilateral Tumoral Involvement and Central Lymph Node Metastasis**

Three articles (23, 25, 28) were analyzed in this section. The articles were heterogeneous (heterogeneity χ^2^= 14.32, *P*=0.001):

RR1= 0.790; 95% CI= 0.547-1.141, RR2= 1.794; 95% CI= 1.327-2.426

RR3= 1.778; 95% CI= 1.314-2.406

Pooled RR= 1.375; 95% CI= 0.836-2.263, *P*=0.210

**Lateral Cervical Lymph Node Metastasis and Central Lymph Node Metastasis**

Three articles (25, 26, 28) were analyzed in this part. Since heterogeneity was seen across the articles (heterogeneity χ^2^= 9.02, *P*=0.011), analysis was done by the random-effect model:

RR1= 3.226; 95% CI= 2.540-4.096, RR2= 4.778; 95% CI= 2.453-9.304

RR3= 2.041; 95% CI= 1.552-2.684

Pooled RR= 2.941; 95% CI= 1.931-4.478, *P*=0.000

**Extrathyroidal Extension and Central Lymph Node Metastasis**

Two articles (22, 24) in this section were not heterogeneous (heterogeneity χ^2^= 1.16, *P*=0.281):

RR1= 2.122; 95% CI= 1.487-3.028, RR2= 1.572; 95% CI= 1.038-2.379

Pooled RR= 1.875; 95% CI= 1.432-2.454, *P*=0.000

**Lymph Node Metastasis and Bad Outcome**

Two articles (29, 30) had suitable criteria for the analysis in this section. After analyzing by the fixed-effect model because of the homogeneity (heterogeneity χ^2^= 0.01, *P*=0.924), these significant outputs were obtained:

RR1= 5.956; 95% CI= 2.566-13.824, RR2= 5.563; 95% CI= 1.706-18.141

Pooled RR= 5.869; 95% CI= 2.890-11.922, *P*=0.000

**Gender and BRAF** ^V600E^ **Mutation**

Two articles (12, 31) were analyzed to assess this relationship. As the articles were heterogeneous (heterogeneity χ^2^= 4.30, *P*=0.038), the random-effect model was used:

RR1= 1.456; 95% CI= 1.246-1.702, RR2= 1.099; 95% CI= 0.881-1.371

Pooled RR= 1.279; 95% CI= 0.968-1.691, *P*=0.084

**Tumor Size and BRAF** ^V600E^ **Mutation**

This relationship was analyzed in two articles (12, 31). As the articles did not show heterogeneity (heterogeneity χ^2^= 0.75, *P*=0.387), analysis was done by the fixed-effect model. The significant results are as follows:

RR1= 1.332; 95% CI= 1.144-1.550, RR2= 1.474; 95% CI= 1.236-1.758

Pooled RR= 1.381; 95% CI= 1.230-1.551, *P*=0.000

**Lymph Node Metastasis and BRAF** ^V600E^ **Mutation**

Three articles (12, 31, 32) were analyzed in this section. According to the heterogeneity of articles (heterogeneity χ^2^= 109.19, *P*<0.001) theses outputs were obtained:

RR1= 3.368; 95% CI= 2.953-3.841, RR2= 1.077; 95% CI= 0.910-1.276

RR3= 1.900; 95% CI= 1.310-2.755

Pooled RR= 1.905; 95% CI= 0.833-4.357, *P*=0.127

**Tumor Stage (TNM) and BRAF** ^V600E^ **Mutation**

Two articles (12, 31) were analyzed in this part. Analysis by the random-effect model on heterogeneous articles (heterogeneity χ^2^= 31.16, *P*<0.001) showed the following non-significant results:

RR1= 1.918; 95% CI= 1.661-2.215, RR2= 1.009; 95% CI= 0.846-1.203

Pooled RR= 1.394; 95% CI= 0.739-2.628, *P*=0.305

**Multifocality and BRAF** ^V600E^ **Mutation**

In the last part of our study, two articles (12, 31) were analyzed. Non-heterogeneous articles (heterogeneity χ^2^= 2.93, *P*=0.087) were analyzed by the fixed-effect model and these non-significant results were obtained:

RR1= 0.970; 95% CI= 0.823-1.144, RR2= 1.179; 95% CI= 0.999-1.391

Pooled RR= 1.037; 95% CI= 0.918-1.171, *P*=0.563

## Discussion

Papillary thyroid microcarcinomas, which are cases of papillary thyroid carcinoma with the greatest diameter of 1 centimeter, represent approximately half of all the cases of this tumor type (10, 11, 19, 20, 23, 28, 29, 33-36). PTMCs account for up to 43% of all thyroid cancers (18). Despite being cancerous, PTMCs have been viewed as tumors with a benign nature that have a minimal clinical impact and do not impact the patient's survival. Nonetheless, mounting evidence indicates that PTMCs exhibit varying degrees of disease severity, and the reported frequencies of the aggressive characteristics can vary greatly (4, 24). 

Previous research findings have demonstrated that this type of tumor is discovered when the thyroid gland is examined microscopically during necropsy or surgery for non-thyroid or non-cancerous thyroid conditions (4, 37). However, in some cases, a papillary microcarcinoma could be the initial tumor that has manifested as a lymph node metastasis. It may also present as a neck mass (17).

The increased rate of detection has been attributed to the broader accessibility of ultrasound and fine-needle aspiration (FNA), likewise the enhanced precision of histopathologic examination of the surgical specimens (17, 18, 29, 35, 37).

In the clinical practice, PTMC, which is a particular subset of PTC, necessitates attention due to its growing prevalence among the PTC patients and its impact on the patient care (26). Therefore, knowing more about this tumor and its characteristics is important. 

Certain PTMCs are thought to be linked to the recurrence of the disease. Despite the development of distant metastasis or even mortality in some cases, a great number of them follow a mild course with a positive prognosis, making the clinical significance of PTMC unclear (12, 23). Majority of the research emphasizes that papillary microcarcinomas typically have a favorable outcome and may exhibit similar behavior to the benign growths (including the potential for the partial spontaneous regression), which could justify a conservative treatment approach. On the other hand, some studies have indicated that microcarcinomas are indeed malignant tumors that may necessitate invasive treatment in certain cases (7). There is an ongoing debate about the clinical significance of papillary thyroid microcarcinoma. While some researchers have observed a benign course with no advancement, others have identified instances of unexpectedly aggressive cancer (29).

When evaluating the factors that influence a patient prognosis, various authors have identified multifocal disease as a negative prognostic indicator. However, age has not consistently been shown to be a predictor of the outcomes for the patients with PTMC (14). Clinicopathological factors that increase the risk of a poor prognosis include advanced age upon diagnosis time, gender, the presence of multiple tumoral foci, LNM, and a high TNM stage. This was expressed in Kwak et al. study (38). In our meta-analysis of factors affecting tumor prognosis, it was shown that lymph node metastasis leads to the bad outcomes of tumors. This relationship was assessed in two articles, both confirming the effect of lymph node metastasis on tumor outcome. The final analysis found that lymph node metastasis could increase the likelihood of poor prognosis six times.

Despite the typically favorable prognosis of PTMC, the disease recurrence after the initial surgical treatment remains a challenging problem (24). Tumor metastasis to lymph nodes results in a higher rate of tumoral recurrence. Furthermore, the current research demonstrated a noteworthy correlation between the stage of the tumor and its likelihood of recurring. Regarding some researchers’ findings, PTMCs larger than 5 mm are more likely to have multifocality, capsule invasion, and lymph node metastasis than those about 5 mm or smaller. However, other studies have found no significant difference in these outcomes (12, 24). Lymph node metastasis was predicted by several factors, including a tumor size of 5 mm or larger, sclerosis, S100A4 expression, extrathyroidal invasion, cyclin D1 expression, and multifocality. Moreover, the occurrence of lymph node involvement in papillary microcarcinomas has been linked to the development of distant metastasis. The latest TNM staging system places greater emphasis on the lymph node metastasis as a determinant of the overall staging compared to the tumor size (17, 32). In a recent extensive case-control study, it was found that patients with lymph node metastasis (LNM) had almost three times higher disease-related mortality rate compared to those without LNM (32). Our meta-analysis showed that extrathyroidal extension and multifocality were correlated with the lymph node metastasis. Both articles analyzed the role of extrathyroidal extension and showed its effect, and pooled RR=2.273 of the analysis was significant. Multifocality was analyzed in two articles, and the RR in both was more than 1; the final pooled RR=1.745 indicated this factor effect on the lymph node metastasis.

The study conducted by Zheng et al. revealed that tumor recurrence was correlated with the extrathyroidal extension (15), the type of surgery performed, and lymph node metastasis (13). However, the recurrence of tumors did not have any connection with the factors of age, gender, multiple foci of the disease, tumor size, advanced stage of the disease (T3/4), and the existence of BRAFV600E mutation (12, 13). Central lymph node metastasis is another factor relevant to the tumor recurrence (26, 39). We identified clinical characteristics predicting recurrent disease: larger tumor size, absence of I-131 therapy, and presence of the lymph node metastasis (14). Age, tumor size, multifocality, lymph node metastasis, gender, and extrathyroidal extension are all recognized as the risk factors for the recurrent disease, as expressed by Yun et al. Conversely, recognized risk factors such as gender, age, and size were found to be associated with only one or, in some cases, none of the extrathyroidal extension and central lymph node metastasis (35). In our meta-analysis of the factors affecting extrathyroidal extension, only gender was a related factor whose association was not statistically significant (P=0.611). Concerning tumor dimensions, the study conducted by Kim et al. demonstrated a correlation between PTMCs larger than 5 mm and tumor recurrence (38). The presence of tumor bilaterality, rather than multifocality, was significantly relevant to the tumor recurrence (37). This study revealed that patients with multiple foci of PTMC had larger tumor diameters and a higher likelihood of experiencing recurrence or persistent disease compared to those with a single focus of PTMC. An advanced clinical stage and larger tumor size independently predicted recurrent or persistent disease. Cases with multiple foci of PTMC and a tumor diameter less than 0.5 cm were observed to present a lower risk of recurrence (30). In our meta-analysis, gender, lymph node metastasis, and extrathyroidal extension were statistically significantly relevant to the recurrence. However, this association was not shown for the age, tumor size, tumor stage, and multifocality. Two articles were assessed for the gender, showing this factor effect on tumor recurrence. One article, which weighed more than the other with RR=3.140 compared to RR=1.322, showed a stronger relationship, but the final result (pooled RR=2.416) confirmed that tumor recurrence was two times more in females than in males. The lymph node metastasis was assessed in four articles, all supporting its relationship with recurrence. According to the final pooled RR, lymph node metastasis increases the risk of tumor recurrence by three folds. Three articles analyzed extrathyroidal extension, showing a relationship between this factor and tumor recurrence. Based on the analysis, pooled RR=6.366 showed that tumor expansion over the thyroid margins remarkably affects tumor recurrence during follow-up.

Other factors analyzed about tumor prognosis were bilateral involvement and central lymph node metastasis (24). The initial involvement of lymph nodes usually starts with those located in the neck central region followed by the nodes in the lateral neck and the superior mediastinum of the neck. Hence, it is crucial to identify the presence of central lymph node metastasis (26). Primary tumors ≥7 mm and multifocality independently predicted PTMC bilaterality, and male gender, age <50 years, and primary tumors larger or equal to 7 mm could be predictors of CLNM. Zhou et al. presented in their study that cases with bilateral PTMC and multifocality may have a higher risk of CLNM, but they were not independent risk factors (23). Despite being clinically undetectable, central lymph node metastasis is a significant risk factor for the recurrence. This research revealed that subclinical CLNM can be predicted by the male sex and the size of the tumor exceeding 7 mm, which were found to be independent factors. However, the variables of lymphovascular invasion, lymphocytic thyroiditis, extrathyroidal extension, multifocality, age, and bilaterality were not considered as predictors of subclinical CLNM (19, 24). Kyung-Eun Kim et al. reported that larger tumor size (>5 mm), bilaterality, extracapsular invasion, and lateral LNM were significantly correlated with the central LNM (26). The presence of positive lateral lymph node metastasis (LNM) was identified as the strongest predictor of positive central LNM (19). The CCLN metastasis was consistently associated with the gender, multifocality, extrathyroidal extension, tumor size, extracapsular invasion, and bilaterality (39). Our analysis showed that gender, tumor size, extracapsular invasion, extrathyroidal invasion, and lateral cervical lymph node metastasis were significantly associated with the central lymph node metastasis. But this relationship was not shown for the age and bilaterality. The gender factor was assessed in six articles, five of which showed its effect on metastasis, but only one paper with RR=0.908 did not show this effect. Finally, pooled RR revealed that central lymph node metastasis in females is two times more than in males. Tumor size was another factor analyzed in seven articles. All of them demonstrated a correlation between this factor and metastasis, and the pooled RR showed that this correlation was twice as strong. The extrathyroidal invasion effect was assessed in five articles. At the end of the analysis, pooled RR showed that central lymph node metastasis is two times more in the patients with extrathyroidal invasion. Finally, after analyzing three articles, lateral cervical lymph node metastasis increased the risk of metastasis by three times.

Papillary thyroid microcarcinomas (PTMCs) may comprise two biologically different subgroups: slow-growing tumors with minimal or no potential for the advancement, and tumors with more aggressive behavior. It is important to differentiate between the two different subpopulations of thyroid nodules, especially before surgery, and finding markers that can do so is crucial for the proper clinical management. In recent years, numerous studies have focused on utilizing genetic and molecular tests on FNAC samples to enhance the precision of cytologic diagnosis of thyroid nodules. One of these factors is the BRAFV600E mutation (40). This mutation is frequent in the late-stage tumors (3 and 4) and those with lymphovascular involvement and metastasis (12, 18). The findings of Zheng study revealed that BRAFV600E mutation had a notable correlation with the male sex, extrathyroidal extension, and LNM; however, no significant correlation was observed with the age, multifocality, TNM staging, or distant metastasis (12). The presence of BRAFV600E mutation in aspiration samples has been linked to the negative prognostic factors, including the larger size of the tumor, extrathyroidal invasion, and a higher TNM stage (21). Kwak and colleagues discovered that there was a significant correlation between BRAFV600E and tumor size (larger than 5 mm), extracapsular extension, and high TNM stage (3 and 4) in individuals diagnosed with PTMC (38, 40). In our analysis, only tumor size had a statistically significant correlation with BRAFV600E mutation (*P*=0.000), but its relationship with the gender, lymph node metastasis, tumor stage, and multifocality was insignificant. Both articles analyzed tumor size relationship with mutation and proved it. In the final analysis, based on the pooled RR, tumor size increased the mutation risk by 1.4 folds.

## Conclusion

Improved diagnostic methods have led to a rise in the occurrence of PTMCs. These tumors may serve as an indicator of the advanced papillary carcinomas. Despite being typically benign and slow-growing, they may sometimes have adverse outcomes, including tumoral distant metastasis and recurrence. Detecting PTMC and monitoring its characteristics offer two advantages: preventing the need for more extensive and prolonged treatments for the advanced tumors and managing the tumor as a distinct ailment.

## Funding

 None.

## Conflict of Interest

None.
